# Editorial: Non-coding RNA in Alzheimer's pathology and diagnosis

**DOI:** 10.3389/fnagi.2022.1030742

**Published:** 2022-09-20

**Authors:** Rajkumar Singh Kalra, Murali Vijayan, Suman Ghosal

**Affiliations:** ^1^Okinawa Institute of Science and Technology, Graduate University, Okinawa, Japan; ^2^Department of Internal Medicine, Texas Tech University Health Sciences Center, Lubbock, TX, United States; ^3^Section on Medical Neuroendocrinology, Eunice Kennedy Shriver National Institute of Child Health and Human Development, National Institutes of Health, Bethesda, MD, United States

**Keywords:** Alzheimer's disease, ncRNAs, lncRNA, ceRNAs, circRNA, ex-miRNAs, miRNA, neurodegenerative diseases

Alzheimer's Disease (AD) is an age-associated neurodegenerative disorder that progressively and irreversibly yields a loss of cognitive function (Masters et al., 2015). Among the multiple theories rooting for the cause of AD, prominent and widely studied ones are the accumulation of amyloid-beta protein and neurofibrillary tangles that disturb cellular and synaptic transmission (Wang et al., 2014; Gouras et al., 2015). Other less-studied theories cite the role of oxidative stress, neuroinflammation, metabolism imbalance, and autophagy-related mechanisms in AD progression (Xu et al., 2015). Despite the decades of developing treatments, current medication is partly successful in easing some AD symptoms. Given the multifactorial nature of AD progression, presently, no treatment is available that could either inhibit or limit the progressive decline. Non-coding RNA (ncRNA) mechanisms in AD recently attracted a lot of attention (Tan et al., 2013). Besides the abundant transfer RNAs (tRNAs) and ribosomal RNAs (rRNAs), recently long (lncRNAs) and small ncRNAs *viz*. microRNAs, competing for endogenous RNAs (ceRNAs), circular RNAs (circRNA), exosome miRNAs (ex-miRNAs), and Piwi-interacting RNA (piRNA) has been at the center of discussion in AD pathology (Doxtater et al., 2020). Single ncRNA can regulate the expression of many genes across the CNS, implying if ncRNA function is dysregulated, it may trigger multiple pathogenic pathways in the brain (Vijayan and Reddy, 2020). Anticipatedly, multiple recent studies have confirmed dysregulated expression of ncRNA's in AD (Millan, 2017). As ncRNA analysis is quicker and cost-effective, its utility in diagnosis may help discriminate between different forms of neurodegenerative disease (Lauretti et al., 2021). Although, recent research on ncRNAs opens a new dimension of knowledge on the mechanisms of AD pathology, there is still much to explore.

In this context, the original research articles and mini/systematic reviews published in the present Research Topic shed light on the role of ncRNAs in AD pathology and provide a larger understanding of the ncRNA mechanisms in AD and their utility for diagnostics and therapeutics.

In an insightful report, Huaying et al. obtained 3,158 lncRNAs by microarray data re-annotation and developed a global network of competing endogenous RNAs (ceRNAs) for AD and normal samples based on their transcriptional profiles. By correlating gene expression data, they identified a total of 255 AD-deficient messenger RNA (mRNA)-lncRNAs. A majority of dysregulated ceRNAs genes were enriched in transcription factors and miRNAs, while the identified miRNA in the lncRNA-mRNA network showed that 40 lncRNA pairs share more than one identified miRNA. Of note, nine lncRNAs were found to be associated with AD, PD, and other neurodegenerative pathologies. More specifically, five lncRNAs were identified to be potential biomarkers for AD. Further, they identified a relative decline in PART1 and an increase in SNHG14 transcript levels in the AD serum samples. Conclusively, they elucidated the role of lncRNAs in the pathogenesis of AD and also the potential utility of new lncRNAs in the diagnostics and therapeutics of AD.

Weighing on the emerging significance of lncRNAs in AD, Asadi et al. in a systematic review, evaluated the association between lncRNAs and AD. Dysregulated functions of lncRNAs in the diverse regulatory pathways in CNS are suspected to play a role in AD pathology that is mainly characterized by the formation of amyloid plaques with the accumulation of β-amyloid and neurofibrillary tangles (NFT) that form as a result of the phosphorylated tau accumulation. To examine this association of lncRNAs in AD, they adopted a six-step strategy and systematically surveyed the published research across seven databases as per the Prisma guideline. Of the 1,591 research reports, 69 articles met the specified inclusion criteria of the original research on AD performed by established molecular techniques. The majority of these reports highlighted the role of BACE1-AS, MALAT1, NEAT1, and SNHG1 lncRNAs in AD, whereas nearly one-third of the reports researched a unique lncRNA. Of note, nearly 56 and 7% of the investigations reported an increase and decline in the lncRNAs levels, respectively. In the line, another review by Zhang et al. comprehensively shed light on the ncRNAs implications in AD and the role of key regulatory pathways in discovering the novel druggable targets in AD. Weighing on ncRNAs therapeutic potential as seen in the preclinical stages of AD (as the earliest feasible druggable targets), they stressed the applications of mimicking or inhibitory ncRNAs in regulating their downstream target mRNAs as interventional therapeutics for AD. The review also highlighted the use of miRNAs and Piwi-interacting RNA (piRNA) as key drug targets for therapeutics in murine models of AD. They underlined the application of circRNAs and lncRNAs with MREs function to work like miRNA sponges that could influence the mRNA-regulating activities of miRNAs. They anticipated a future focus on sensitive RNA detection assays to ascertain the utility of key ncRNAs for preclinical or diagnostic applications for AD.

The function of circRNAs, a novel type of endogenous ncRNA is implicated in regulating gene expression in mammals. Recent studies revealed the relevance of circRNAs with neurological diseases, including AD. To gain further insight into it, in original research, Liu et al. identified an aberrant circRNA, *viz*. hsa_circ_0003391, that exhibited a significant downregulation in the peripheral blood of AD patients. To evaluate the clinical manifestation of hsa_circ_0003391 in AD, a receiver operating characteristic (ROC) curve analysis was performed to assess its potential diagnostic value, which was found to be statistically significant [area under the curve (AUC) value: 0.7283]. Bioinformatics approaches further predicted miR-574-5p to be a potential hsa_circ_0003391 target, which exhibited an anticipated increase in the AD groups. These results hinted at a potential correlation of hsa_circ_0003391 expression with clinical manifestations of AD. Overall, this report suggested a potential relationship of decreased hsa_circ_0003391 levels in the peripheral blood with AD and hinted at the development of novel AD therapeutics by targeting ncRNA. In this line, a mini review by Dong et al. assessed research on exosome miRNAs (ex-miRNAs) that function in clinical dementia and examined the potency of ex-miRNAs as an early diagnostic biomarker for common or AD-related dementia. They highlighted the necessity of a reliable biomarker for the cerebrospinal fluid (CSF) and peripheral blood that may enable early clinical diagnosis of dementia. Given the susceptibility of interference by several factors in the peripheral circulation to circulating miRNA, they argued the suitability of ex-miRNAs for diagnosis, citing their greater stability.

In another comprehensive review report, Samadian et al. conferred recent evidence emphasizing the role of miRNAs in the development of AD. The review carefully assessed the role of several miRNAs, *viz*. miR-195, miR-200a-3p, miR-338-5p, miR-125b-5p, miR-34a-5p, miR-132, miR-339-5p, miR-384, miR-425-5p, miR-135b, and miR-339-5p, those were shown to have a function in the development of AD through their interaction with BACE1. Several other miRNAs were suggested to impact the inflammatory responses in AD progression. This review amply covers new knowledge on the aberrant expression of miRNAs in the plasma of AD subjects, aptitude for differentiation, and AD-modifying agents that affect miRNA profiles *in vitro* or *in vivo* models.

In conclusion, this Research Topic collects evidence highlighting the relevance of ncRNAs in AD pathology and its progression. It specifically conferred the diverse role that lncRNAs, microRNAs, ceRNAs, circRNA, ex-miRNAs, piRNA play in AD pathology and may also pave the way for its diagnosis and therapeutics in clinics.

## Author contributions

All authors listed have made a substantial, direct, and intellectual contribution to the work and approved it for publication.

## Conflict of interest

The authors declare that the research was conducted in the absence of any commercial or financial relationships that could be construed as a potential conflict of interest.

## Publisher's note

All claims expressed in this article are solely those of the authors and do not necessarily represent those of their affiliated organizations, or those of the publisher, the editors and the reviewers. Any product that may be evaluated in this article, or claim that may be made by its manufacturer, is not guaranteed or endorsed by the publisher.
